# One-pot synthesis of zinc oxide nanoparticles via chemical precipitation for bromophenol blue adsorption and the antifungal activity against filamentous fungi

**DOI:** 10.1038/s41598-021-87819-2

**Published:** 2021-04-15

**Authors:** Kovo G. Akpomie, Soumya Ghosh, Marieka Gryzenhout, Jeanet Conradie

**Affiliations:** 1 Department of Chemistry, University of the Free State, Bloemfontein, South Africa; 2 Department of Pure & Industrial Chemistry, University of Nigeria, Nsukka, Nigeria; 3 Department of Genetics, University of the Free State, Bloemfontein, ZA9300 South Africa

**Keywords:** Environmental sciences, Chemistry, Engineering, Materials science

## Abstract

In this research, zinc oxide nanoparticles (ZnONPs) were prepared via a facile one-pot chemical precipitation approach and applied in the adsorption of bromophenol blue (BRB) and as antifungal agents against the filamentous fungi and plant pathogens; *Alternaria alternata* CGJM3078, *Alternaria alternata* CGJM3006 and *Fusarium verticilliodes* CGJM3823. The ZnONPs were characterized by the UV–Vis, FTIR, XRD, TGA, BET, SEM, TEM, and EDX techniques, which showed efficient synthesis. The characteristics ZnO UV–Vis absorption band was observed at 375 nm, while the XRD showed an average ZnONPs crystalline size of 47.2 nm. The SEM and TEM images showed an irregular shaped and aggregated porous structure of 65.3 nm average-sized ZnONPs. The TGA showed 22.9% weight loss at 800 °C indicating the high thermal stability of ZnONPs, while BET analysis revealed a surface area, pore volume and pore diameter of 9.259 m^2^/g, 0.03745 cm^3^/g and 9.87 nm respectively. The Freundlich, pseudo-second-order, and intra-particle diffusion models showed R^2^ > 0.9494 and SSE < 0.7412, thus, exhibited the best fit to the isotherm and kinetics models. Thermodynamics revealed feasible, endothermic, random, and spontaneous adsorption of BRB onto the synthesized ZnONPs. The antifungal assay conducted depicts strong antifungal activities against all three tested fungi. Noticeably, ZnONPs (0.002–5 mg/mL) showed maximum activities with the largest zone of inhibition against *A*. *alternata* CGJM 3006 from 25.09 to 36.28 mm. This was followed by the strain *F*. *verticilliodes* CGJM 3823 (range from 23.77 to 34.77 mm) > *A. alternata* CGJM3078 (range from 22.73 to 30.63 mm) in comparison to Bleach 5% (positive control). Additionally a model was proposed based on the possible underlying mechanisms for the antifungal effect. This research demonstrated the potent use of ZnONPs for the adsorption of BRB and as effective antifungal agents.

## Introduction

In recent years, there have been growing concerns arising from the rapid pollution of water bodies around the world^1^. This is attributed to technological advancements resulting in the rapid growth of industries, and subsequently the pollution of water from industrial effluents^2,3^. This water pollution has significantly affected aquatic lives, made the water unsafe for drinking, and resulted in several health effects^4,5^. Among several water pollutants, dyes have gained significant attention from environmental scientists^6^. This is because dyes are extensively used in the textile, leather, cosmetic, and paper industries^7^. The textile industry alone consumes over 700,000 tons of dyes annually^8,9^. Thus, there is a high amount of dyes in the effluents released from these industries. Apart from that, dyes are allergenic, mutagenic, and carcinogenic at certain concentrations, hazardous, affect photosynthesis in marine organisms, and are resistant to biodegradation^10^. Therefore, the treatment of dye-polluted water is important.

Bromophenol blue (BRB) dye is widely used as a laboratory indicator and in the textile and printing industries^8,11^. However, most studies have focused on the treatment of water contaminated with dyes such as methylene blue, congo red, and malachite green, but only a few studies are documented the removal of BRB despite its harmful nature in the environment^12^. Hence, this research considers the removal of BRB from water.

Several methods have been utilized over the years for the treatment of dye-polluted water such as filtration, coagulation, solvent extraction, precipitation, adsorption, ion exchange, floatation, biological treatment, catalytic degradation, ozonation, and oxidation^13,14^. Adsorption is the most efficient and promising method but is limited by the high cost of activated carbon used as a commercial adsorbent for water treatment^9,15^. Therefore, other efficient low-cost adsorbents such as bio-waste, natural inorganic materials, polymeric materials, and nanomaterials have been harnessed as suitable alternatives to activated carbon^10,16–21^.

Among the alternative adsorbents, nanomaterials have gained tremendous attention in recent years due to their high efficiency, high surface reactivity, broad application for several purposes, and environmental compatibility^8,17,22^. Metallic nanoparticles are known to be promising owing to their efficient adsorptive and catalytic degradation potentials for dye containing water^23–25^. In particular, zinc oxide nanoparticles (ZnONPs) have received significant attention for water treatment because of their nontoxicity, long-term stability, acceptable cost, biocompatibility, surface properties and potent antimicrobial activities against microbes frequently encountered in water^26–28^. Thus, a combination of the adsorption potentials and the antimicrobial activities in water treatment would be highly effective to obtain portable water. Therefore, the efficient treatment of dye-polluted water by the use of ZnONPs is well documented^26,29,30^. However, from a thorough literature search, there is no evidence of the use of ZnONPs in the adsorption of BRB from water. Thus, this study evaluated for the first time the potentials of ZnONPs in the treatment of BRB polluted water.

Several studies have been performed on the antibacterial and antifungal activities of ZnONPs, which proved that ZnONPs are potent antimicrobial agent^27,31–35^. However, studies on the antifungal activity of nanoparticles against filamentous fungi are rare^36,37^. In this study, the two plant pathogens were studied. The filamentous fungus *Alternaria alternata* is a pathogen of fruit and vegetables such as strawberries, tangerines, mandarins, grapefruits, and tomatoes, thus causing extensive post-harvest spoilage and loss^38,39^. Additionally, *A. alternata* employs cell wall degrading enzymes, such as pectic enzymes, and organic acids that lower the pH and act synergistically with the enzymes to digest calsium-acetate, which causes damage to the host tissues^38^. In humans, *Alternaria alternata* is associated with hypersensitivity pneumonitis, bronchial asthma, allergic sinusitis, rhinitis, and cutaneous, subcutaneous infections in humans^40^.

The filamentous fungus *Fusarium verticilliodes* is a seed-borne endophytic pathogen of maize and cereals such as wheat, and can infect a wide variety of other plants worldwide^41,42^. Besides, *F. verticilliodes* secretes fumonisin B that accumulates in the kernels of maize or wheat, causing toxicity in resulting food commodities^43,44^. *Fusarium* mycotoxin adulteration in agricultural commodities poses a global threat to food safety and has substantial economic significances^45,46^.

There is a need to discover novel control agents to prevent plant diseases and post-harvest losses of food crops and grains associated with filamentous fungi, as well as to minimize harmful effects of these fungi on humans and livestock. Although a few studies have reported ZnONPs antifungal activities against *Alternaria alternata*^36,37^, no study was conducted specifically on the two strains (CGJM3078 and CGJM3006) of *Alternaria alternata*. Besides, no antifungal studies with ZnONPs were conducted so far against strains of *F. verticilliodes*. The present study investigated the extent of the antifungal activities of ZnONPs against these economically significant postharvest fungal pathogens.

The aim of this study is the evaluation of the adsorptive potentials of ZnONPs for BRB dye as well as the antifungal activity against *A. alternata* and *F. verticilliodes*. The ZnONPs were synthesized by a simple traditional one-pot chemical precipitation approach and characterized. The synthesized ZnONPs were then utilized for BRB adsorption. Finally, the antifungal activity of the synthesized ZnONPs against *A. alternata* and *F. verticilliodes* was investigated in vitro, and a possible mechanism of action against these fungi was proposed.

## Materials and methods

### Chemicals used

Zinc acetate dihydrate (Zn(CH_3_COO)_2_·2H_2_O), bromophenol blue (C_19_H_10_Br_4_O_5_S), sodium hydroxide (NaOH), nitric acid (HNO_3_), and hydrochloric acid (HCl) were purchased from Sigma-Aldrich, South Africa. All the chemicals were used as purchased without any purification.

### Synthesis of ZnONP

A simple one-pot synthesis via chemical precipitation was utilized for the preparation of ZnONPs^47^. Herein, 4.0 g of zinc acetate was added to 100 mL of distilled water in a beaker. The solution was stirred with a magnetic stirrer on a hot plate at 30 °C for 40 min. This was followed by the dropwise addition of 0.2 M NaOH to the solution with continuous stirring until the pH was 11.0. The solution became white due to the precipitation of ZnO and Zn(OH)_2_, the solution was stirred further for 1 h, after which it was allowed to stand for 50 min. The filtrate was decanted and the synthesized precipitate was washed repeatedly with excess distilled water until neutral pH and finally with ethanol. The precipitate was further dried in an oven at 250 °C for 5 h in order to decompose any Zn(OH)_2_ into ZnONPs. The as-prepared ZnONPs were stored in an airtight sample container and kept in a desiccator until use.

### Characterization of the synthesized ZnONPs

The ZnONPs were characterized to evaluate the surface properties necessary for efficient adsorption of BRB from water. The field emission scanning electron microscopy (FE-SEM; Jeol model) was used to examine the particle size and morphology, while the Energy-dispersive X-ray spectroscopy (EDS; Oxford model) coupled to the SEM instrument was used to determine the elemental composition. The morphology and surface structure was further analyzed with the Transmission electron microscopy (TEM) (Philips-FEI-CM100 model) equipped with a Mega View III digital camera. The functional groups of ZnONP were examined by the Fourier transform infrared (FTIR) spectroscopy (FTIR; Bruker Tensor 27 model). The crystalline phases were identified by the X-ray diffractometer (XRD; Brucker model) with Cu radiation of 1.5 Å at the 2-theta range of 10°–80°. The pH drift method was used to evaluate the pH point of zero charge (pHpzc) as described elsewhere^48^. The UV absorption spectra of the ZnONPs were obtained using distilled water as a reference with the UV-Spectrophotometer (Shimadzu UV-1800 model) in the range 250 to 850 nm. Thermal stability was analyzed by the thermo-gravimetric analyzer (TGA; Mettler Toledo Model). The surface area analyzer (Micrometrics ASAP 2020 model) was used to examine the Surface area (S_BET_) and pore properties and the results were refined by the MicroActive VI.01 software.

### Adsorption removal

A stock solution of BRB was made by dissolving 50 mg of C_19_H_10_Br_4_O_5_S in a 500 mL volumetric flask to make a concentration of 100 mg/L. Serial dilution of the stock was done to obtain lower concentrations of 10–50 mg/L. The pH of the solution was adjusted from 2.0 to 9.0 using 0.1 M NaOH and HCl. Batch adsorption was applied to determine the influence of solution pH, ZnONPs dosage, BRB concentration, sonication time, and temperature at the operating conditions presented in Table 1. Batch BRB adsorption was carried out by adding a given amount of ZnONPs to 10 mL of a given BRB concentration at the specified pH. The mixture was sonicated at a particular temperature in an ultrasonic 2.5 L water-filled bath at the specified time. At the end of the given time, the solution was centrifuged at 8000 rpm for 30 min, and the filtrate was analyzed for residual BRB at a maximum wavelength of 590 nm, using the UV spectrophotometer (Shimadzu UV-1800 model). The percentage adsorption and uptake capacity q_e_ (mg/g) were calculated from the percentage removal and mass balance equations, respectively^8^:

**Table 1:** Effect of various parameters and operating conditions on the adsorption of bromophenol blue onto ZnONPs.

Parameters	Range studied	Conditions maintained
Solution pH	2, 3, 4, 5, 6, 7, 8, 9	Dosage 0.1 g, dye conc 50 mg/L, time 180 min, temp 300 K
ZnONP dosage (g)	0.1, 0.15, 0.2, 0.25, 0.3	pH 4.0, dye conc 50 mg/L, time 180 min, temp 300 K
Dye concentration (mg/L)	10, 20, 30, 40, 50	pH 4.0, dosage 0.1 g, time 180 min, temp 300 K
Sonication time (min)	5, 10, 20, 40, 60, 80, 100, 120, 140, 160, 180	pH 4.0, dosage 0.1 g, dye conc 50 mg/L, temp 300 K
Temperature (K)	300, 306, 313, 318, 323	pH 4.0, dosage 0.1 g, dye conc 50 mg/L, time 180 min

1 $$Adsorption (\%) =\left(\frac{\left({C}_{o}-{C}_{e}\right)}{{C}_{o}}\right)*100$$

2 $${q}_{e}=\frac{\left({C}_{o}-{C}_{e}\right)V}{m}$$

where *C*_*e*_ and *C*_*o*_ in mg/L are the final and initial concentrations of BRB in solution, respectively. *m* (g) is the mass of ZnONPs used and *V* (L) is the volume of BRB solution used.

### Isotherm, kinetic and thermodynamics of adsorption

The isotherm modeling on the adsorption of BRB onto ZnONPs was evaluated from the Freundlich, Temkin, Langmuir, and Flory–Huggins models ^12^. The adsorption kinetics was deduced from the pseudo-first-order (PFO), pseudo-second-order (PSO), liquid film diffusion (LFD), and intraparticle diffusion (IPD) rate models, while thermodynamics was deduced from Van’t Hoff’s Equation ^49^. The equation and symbols of the isotherm, kinetics, and thermodynamics are described in the Supplementary Information.

### Statistical analysis

Each experiment was done in duplicate and the average value was computed. The error bars in the figures indicate the standard deviation from the mean. The statistical function of the origin 2019b software was used to determine the sum square error (SSE) and the coefficient of determination (R^2^), used to analyze the best-fitted isotherm or kinetic model.

### Antifungal analysis

The in vitro antimicrobial activities of the ZnONPs were tested against the three fungal strains, viz* A. alternata* CGJM3078, *A. alternata* CGJM3006, and *F verticilliodes* CGJM3823 fungi [CGJM = Cultures Gert Johannes Marais]. The fungal strains were procured from Dr. Gert Marais, Department of Plant Sciences, University of the Free State, Bloemfontein, South Africa. The antimicrobial activity screening was performed with the well diffusion technique^50,51^ on malt agar (Malt extract 2%; Dextrose 2%; Agar 2% Biolab, Merck, Johannesburg) media plates. For the technique, a pinch amount of mycelia scraping from each of the fungi was inoculated in 15 mL of Malt extract dextrose broth (Malt extract 2%; Dextrose 2%) and cultivated for 3 days at 25 °C. Following the incubation, the cultures were adjusted to a concentration of 10^6^ cfu/mL (cfu—colony forming units), inoculated in molten Malt Extract Agar, swirled gently, and poured into 90 mm Petri plates (Ascendis, Johannesburg). The inoculated plates were dried under a Bio-Safety Cabinet Class II (ESCO Technologies Pty Ltd, Gauteng, South Africa).

Following air-drying, the solidified agar media plates were bored (bore size = 5 mm) with wells with a metallic plug borer and filled with 40 µL of the ZnONPs made into twofold serial dilutions (0.002, 0.004, 0.009, 0.019, 0.039, 0.078, 0.156, 0.312, 0.625, 1.25, 2.5, 5) mg/mL, in accordance to a previous study^33^. Bleach disinfectant [Ca(ClO)_2_] (5%) served as the positive control while sterile distilled water was used as the negative control for all the experiments. Plates were incubated at room temperature in the upright position until zones of inhibition were observed, which was considered an endpoint parameter for the antimicrobial activities. The diameter of the inhibition zones was measured using the Image J software program (https://imagej.nih.gov/ij/) as per a previous study^51^. All the culture media were procured from Sigma Aldrich, Merck KGaA, Darmstadt, Germany, and Neogen Culture media, Heywood, UK respectively.

## Results and discussion

### Characterization of ZnONP

**Figure 1:**
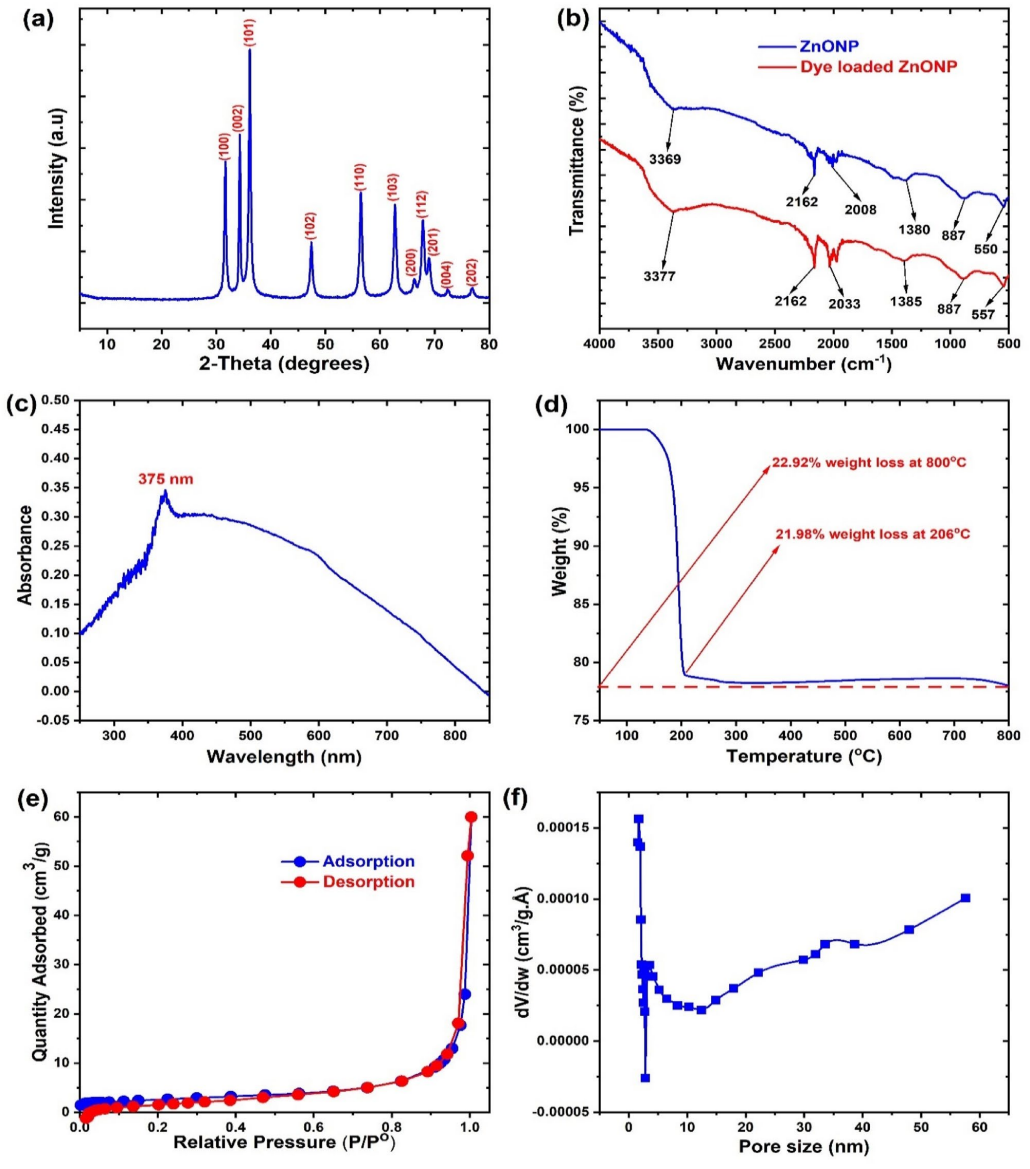
The (**a**) X-ray diffraction (**b**) Fourier transform infrared (**c**) Ultraviolent spectra **(d**) Thermogravimetric analysis (**e**) Nitrogen adsorption–desorption isotherm and (**f**) Pore analysis of the synthesized ZnONPs.

The XRD characterization used to examine the crystalline phases of ZnONPs is shown in Fig. 1a. The spectrum showed diffractions patterns at 2θ values of 31.69°, 34.33°, 36.11°, 47.43°, 56.49°, 62.76°, 66.23°, 67.88°, 68.97°, 72.37° and 76.93°, which conforms to the hexagonal ZnO crystalline planes of (100), (002), (101), (102), (110), (103), (200), (112), (201), (004) and (202) respectively, as indexed in the standard JCPDS card no. 36-1451^26^. This confirms the successful synthesis of ZnO. The XRD result is similar to that reported in the green synthesis of ZnONPs using the extract of stevia^52^. In addition, the as-prepared ZnONPs had high crystalline structure as revealed by the high intensity of the diffraction peaks. Apart from that, the fact that no other diffraction peak was observed in the XRD suggests pure synthesized ZnONPs. The average nanoparticle crystalline size of ZnONPs as deduced from the Debye–Scherrer’s equation was 47.2 nm using the characteristic peak at 36.11°. This is similar to the ZnONPs sizes in the range of 40.3–49.3 nm obtained from the biosynthesis using the extract of *Prosopis farcta* at different concentrations of the precursor (ZnSO_4_·7H_2_O)^53^. Besides, the size was larger than 15.41 nm and 5–15 nm reported in the green synthesis of ZnONPs using aqueous extracts of *Deverra tortuosa*^54^ and *Mussaenda frondsa*^55^ respectively.

The FTIR spectra of ZnONPs before and after the adsorption of BRB are shown in Fig. 1b. The spectra help to identify the functional groups present on ZnONPs. The band at 3369 cm^−1^ corresponds to the O–H stretching vibration, while the O=C=O functionality of absorbed CO_2_ on the ZnONPs was depicted by the bands at 2162 cm^−1^ and 2008 cm^−1^^56^. The absorption band at 1380 cm^−1^ corresponds to the OH bending of water acquire from the absorption of moisture from air^26^. Also, the Zn–O stretching vibrations were indicated by the bands at 887 cm^−1^ and 550 cm^−1^, which confirms the successful synthesis of the ZnONPs. After the adsorption of dye, the BRB-loaded ZnONPs showed shifts in absorption bands from 3369 to 3377 cm^−1^ and 1380 to 1330 cm^−1^ for OH, from 2008 to 2033 cm^−1^ for the O=C=O, and from 550 to 557 cm^−1^ for the Zn–O functionalities. This indicated the involvement of the OH and Zn–O groups in the uptake of BRB on the adsorbent and that the mechanism of BRB uptake on ZnONPs could be attributed to electrostatic interactions, H-bonding as well as weak Vander Waals interaction^47^.

The UV spectrum of the as-prepared ZnONPs is shown in Fig. 1c. The characteristic ZnO peak was observed at 375 nm, this corroborates the efficient and successful synthesis of ZnONPs. The absorption at 375 nm is attributed to the intrinsic bandgap of ZnO from the valence to the conduction bands resulting from the electronic transitions^57^. Similar UV absorption was achieved at 374 nm for the green synthesis of ZnONPs using the aqueous extract of *Deverra tortuosa*^54^. Also, Zare et al.^58^ showed a similar UV absorption of green synthesized ZnONPs in the range of 376–360 nm, while absorptions at 350–370 nm was reported by Khatami et al.^59^. The thermal stability of the synthesized ZnONPs was evaluated from the TGA as presented in Fig. 1d. Significant weight loss was observed from 98 to 206 °C, which could be attributed to the removal of absorbed water, CO_2,_ as well as other low-temperature components from the synthesized ZnONPs. At 800 °C, the ZnONPs showed a weight loss of 22.92% indicating high thermal stability of 78.98%. The as-prepared ZnONPs demonstrated efficient thermal stability in comparison to that prepared by Yuvaraja et al.^29^, which showed over 35% weight loss up to 450 °C. This indicated the efficiency of our synthetic procedure in the preparation of ZnONPs with enhanced thermal characteristics for higher temperature applications.

The nitrogen adsorption–desorption isotherm (Fig. 1e) of the ZnONPs as obtained from BET analysis revealed a surface area of 9.259 m^2^/g and correlated with type III isotherm according to the IUPAC classification^47^. Also, the pore characteristics (Fig. 1f) as obtained from the Barret-Joyner-Halenda (BJH) method showed a pore volume of 0.037453 cm^3^/g and an average pore diameter of 9.87 nm indicating the mesoporous structure. Although the surface area of our as-prepared adsorbent was lower than 26.78 m^2^/g obtained from the hydrothermal synthesis of ZnONPs used in the adsorption of heavy metals^26^, the mesoporous structure (see discussion in the next paragraph) would be highly influential for the efficient removal of dye molecules from solution^60^.

**Figure 2:**
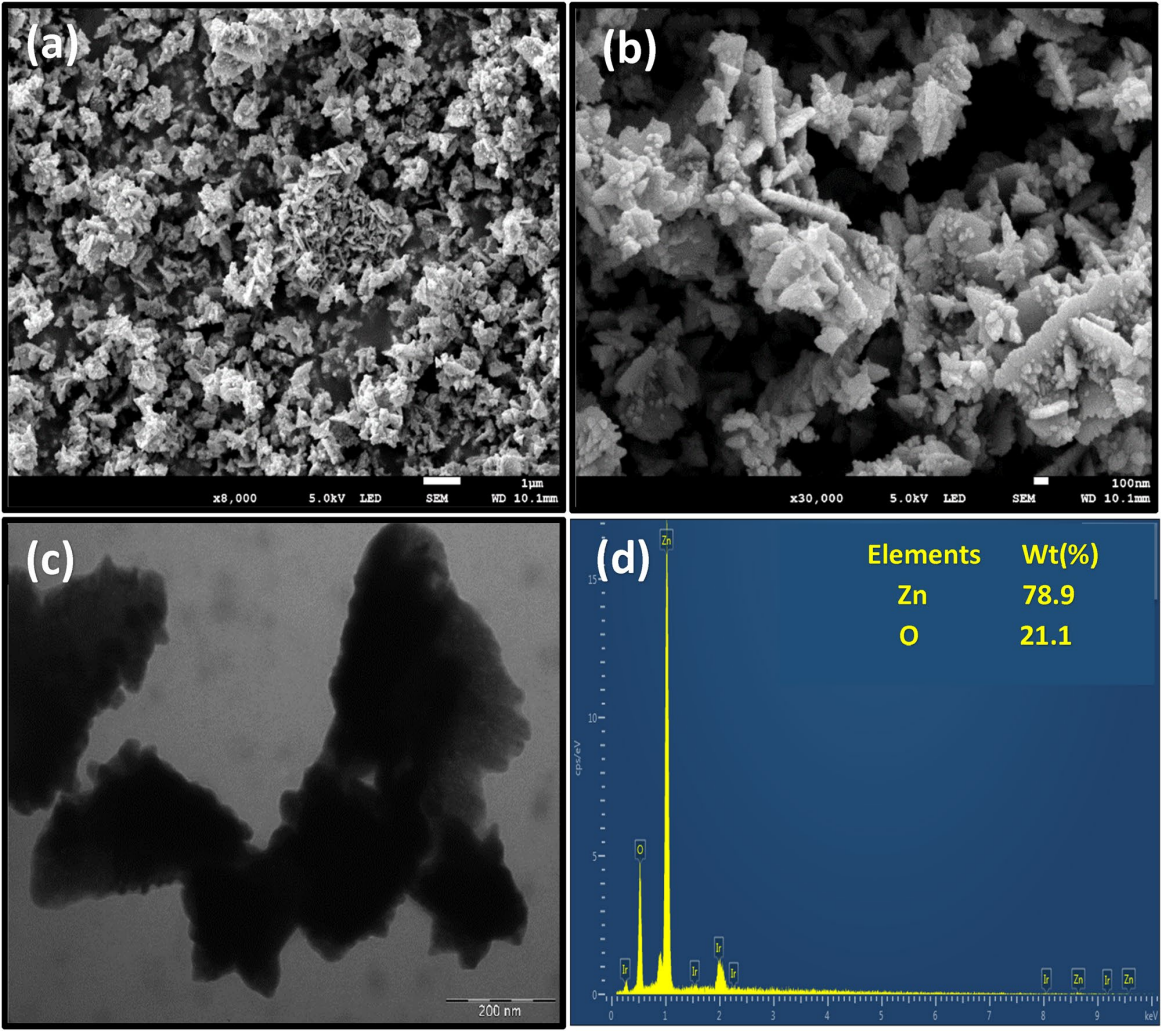
The (**a**, **b**) Scanning Electron microscopy (**c**) Transmission electron microscopy and (**d**) Energy dispersive X-ray spectrum of the synthesized ZnONPs.

The SEM images of the as-prepared ZnONPs are shown in Fig. 2a and b. As observed, the ZnONPs adsorbent is associated with an irregular surface structure, particle aggregation, with a highly porous morphology, which corroborates the porosity measurements. The average ZnONPs size of 65.3 nm was obtained from the SEM structure. This lies in the size range of 60–72 nm obtained in the hydrothermal synthesis of ZnONPs^61^. Again, the porous structure of the synthesized ZnONPs would ensure easy diffusion of dyes molecules favoring efficient adsorption of the pollutant from solution^62^. The agglomerated characteristics of the synthesized ZnONPs were further verified from the TEM image (Fig. 2c). Besides, the EDX spectra (Fig. 2d) of the adsorbent, showed 78.9% zinc and 21.1% oxygen, which further confirms the successful synthesis of the ZnONPs. The absence of other elements also indicates a pure synthesized ZnONPs, which corroborated the results of the XRD analysis. A similar finding was also reported by other workers^26,63,64^. The absence of impurities in the synthesized ZnONPs is important for efficient antimicrobial activity^64^.

### BRB uptake on ZnONPs

**Figure 3:**
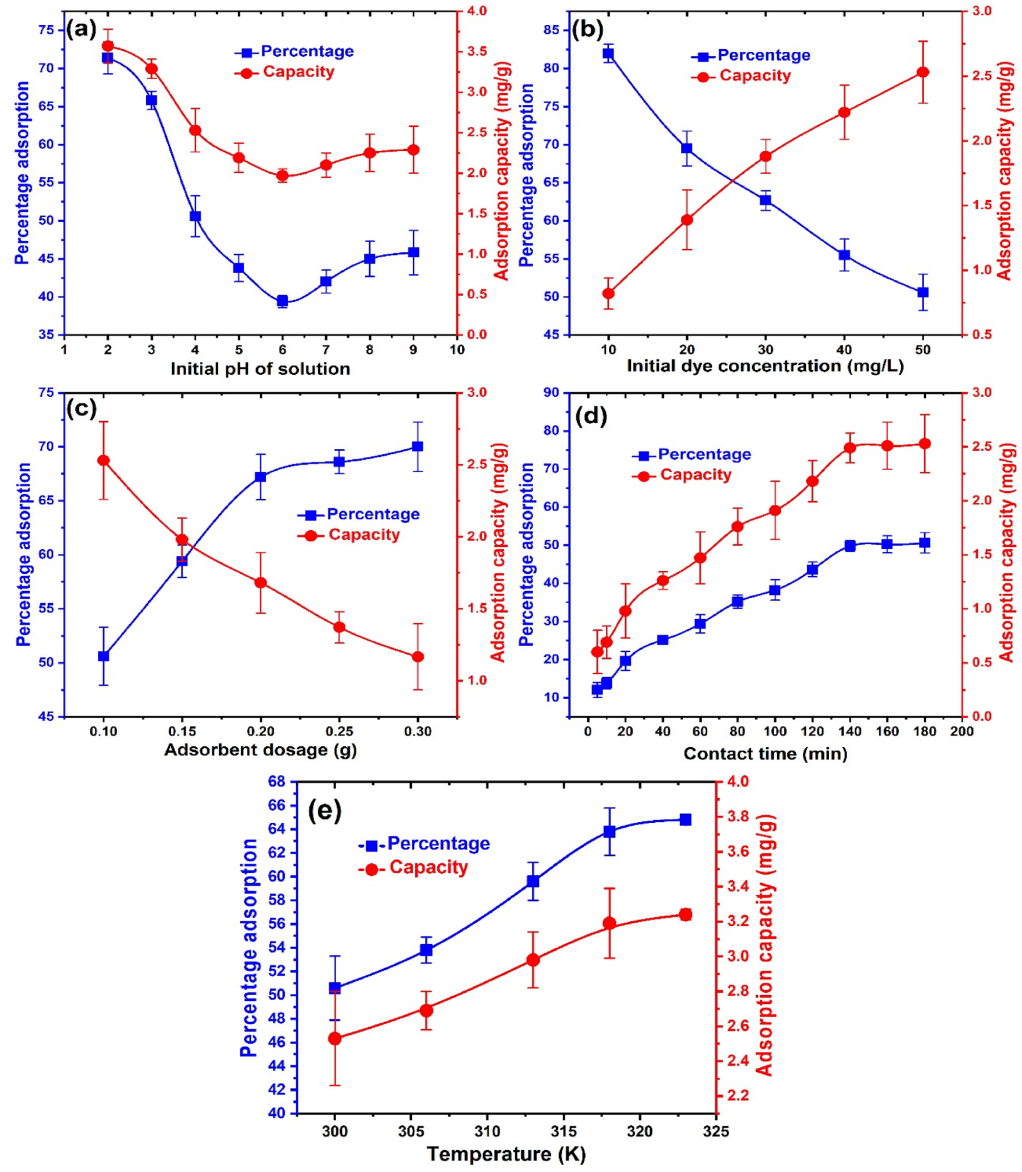
The influence of (**a**) solution pH (**b**) BRB concentration (**c**) ZnONPs dosage (**d**) contact time and (**e**) solution temperature on the removal of BRB onto ZnONPs.

Several operating parameters such as the temperature of the solution, dye concentration, solution pH, time, and material dosage are known to influence the overall uptake of pollutants onto adsorbent materials^17,65,66^. Thus, we investigated the effect of these parameters on the uptake of BRB onto ZnONPs. Figure 3a illustrates the influence of pH on dye removal from solution onto the as-prepared particle. A steady decrease in the adsorbent’s adsorption capacity and percentage uptake of BRB from pH 2.0 to 6.0 was obtained after which a slight increase up to pH 9.0 was observed. This trend is strongly dependent on the pHpzc of ZnONPs as well as the pKa of BRB dye in solution. The pHpzc is the pH at which the net surface charge on the adsorbent is zero^67^. Usually the adsorbent surface is positively or negatively charged at pH values below and above the pHpzc, respectively^68^. The pHpzc of the synthesized ZnONPs was 6.3 while BRB has a pKa value of 3.84^8^. Thus, ZnONPs are associated with a positive surface charge at pH values below 6.3 but becomes negative above this pH. Also, BRB exists as negatively charged molecules below 3.84 after which it becomes neutral and then exists as positively charged species as pH increases. Thus the optimum uptake achieved at pH values below 4.0 was due to strong electrostatic attraction between the positive ZnONPs surfaces and the anionic BRB species in solution. Again, the slight increase observed from pH 6.0 to 9.0 is probably due to the attraction between positively charged BRB species in solution and the negatively charged adsorbent surface. Although optimum BRB removal was obtained at pH 2.0, this pH is too acidic to be associated with real dye-polluted wastewaters. Therefore, we selected pH 4.0 for subsequent experiments due to its closer association with real dye polluted water and the higher uptake recorded compared to values from pH 5.0 to 9.0.

The influence of the initial concentration of dye on the removal of BRB onto ZnONPs is shown in Fig. 3b. With the increase in BRB concentration from 10 to 50 mg/L, an increase in the adsorption capacity of ZnONPs from 0.82 to 2.53 mg/g and a decrease in percentage adsorption from 82.1 to 50.6% was displayed. This trend is consistent with the findings of other researchers^26,69,70^. Thus as the BRB concentration in the aqueous phase increased, more BRB molecules were fixed on the active sites of ZnONPs^71^, which resulted in site saturation, surface repulsions, and less percentage removal of dye from solution^72^. The BRB concentration of 50 mg/L was selected for the batch experiments due to the associated optimum adsorption capacity, which indicated effective utilization of the actives sites of the as-prepared ZnONPs. On the other hand, the effect of ZnONPs dosage on the removal of BRB on the adsorbent displayed an opposite trend to that of the initial dye concentration. As observed from Fig. 3c, with increasing dosage of ZnONPs from 0.1 to 0.3 g, a decrease in the adsorption capacity from 2.53 to 1.17 mg/g and an increase in the percentage adsorption of BRB from 50.6 to 70.2% was recorded. Since the BRB concentration in solution was constant at 50 mg/L, as the dosage of ZnONPs was increased, there was a corresponding increase in the active sites, thus accounting for the higher uptake of BRB from solution^62,73,74^. This in turn resulted in less saturation and less efficient use of the active sites of ZnONPs due to decreasing BRB concentration in solution^5,75^. Hence, we utilized ZnONPs dosage of 0.1 g to enable optimum use of the active sites of the adsorbent^68^.

Figure 3d relates the influence of time on the removal of BRB onto ZnONPs. A steady increase in both the percentage adsorption and adsorption capacity with an increase in time from 5 to 140 min was obtained. Thereafter, there was no noticeable change in the uptake of BRB onto ZnONPs indicating that equilibrium was reached^76^. At the initial stages of adsorption, the ZnONPs sites were vacant and BRB concentration in solution was high^49^. This prompted a spontaneous interaction between the vacant sites and BRB molecules resulting in the initial rapid uptake. The interaction decreased with time due to the occupation of the sites of ZnONPs as well as the reducing dye concentration in solution. Eventually equilibrium was achieved, attributed to the saturation of the sites of ZnONPs^13^. A similar result was obtained in the abstraction of phenol from pharmaceutical and synthetic industrial wastewater onto ZnONPs^77^. We maintained an adsorption time of 180 min to enable equilibrium in the uptake of BRB from the solution. Furthermore, the influence of solution temperature on the removal of BRB onto the synthesized ZnONPs is illustrated in Fig. 3e. With an increase in solution temperature from 300 to 323 K, an increase in ZnONPs adsorption capacity from 50.6 to 64.8%, and percentage adsorption of BRB from 2.53 to 3.24 mg/g were achieved. This suggests that the uptake of BRB on ZnONPs is likely an endothermic one since it is favored at higher temperatures. The improved BRB uptake at higher temperatures could be attributed to enhanced interaction between the BRB species in solution and the adsorption sites of ZnONPs prompted by higher kinetic energy overcoming mass transfer resistances^78^. A similar finding was also documented in the uptake of As (III) from solution on ZnO nanorods^29^.

### Isotherm analysis of BRB adsorption

**Figure 4:**
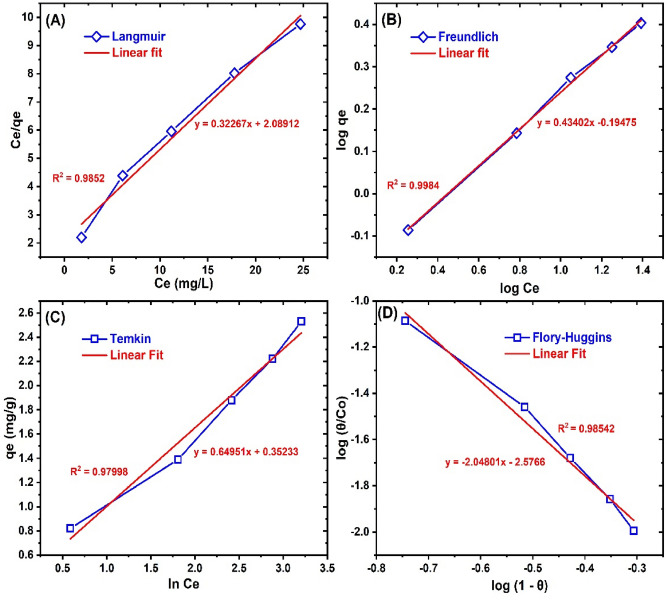
The (**A**) Langmuir (**B**) Freundlich (**C**) Temkin and (**D**) Flory–Huggins isotherm plots for the adsorption of BRP onto ZnONPs.

The adsorption isotherm modeling of BRB adsorption on the as-prepared ZnONPs was conducted to obtain useful information on the favorability of the adsorption process, nature of adsorption as well as potent interaction between the two phases^62,79^. This was analyzed by the Langmuir, Temkin, Freundlich, and Flory–Huggins models^80^ as illustrated in Fig. 4. The calculated isotherm parameters obtained from the modeling are presented in Table 2. The best model fit was judged by the closer the R^2^ value is to one and the smaller SSE values. The Langmuir model, which depicts a monolayer dye uptake on a homogenous material surface^49^, presented a lower R^2^ and higher SSE than the Freundlich model and thus was not applicable in the description of BRB adsorption onto ZnONPs. The Freundlich model gave the best fit to the process, presenting the highest R^2^ of 0.9984 and lowest SSE of 0.0002. This implies a multilayer BRB uptake on a heterogeneous ZnONPs surface and that physisorption must have played a major role in the overall removal process^5^. This result was not consistent with the report of other researchers on the adsorption of heavy metals, phenol and, dyes onto ZnONPs^26,29,30,77,81–83^. They deduced that the Langmuir model was more appropriate indicating a monolayer uptake of the pollutants on ZnONPs. However, our result corroborated the report by Khosla et al.^84^, that the Freundlich model presents a superior fit in the adsorption of anionic dyes (such as BRB) onto ZnONPs. Moreover, a good affinity between the adsorbent and pollutant in solution is usually indicated by the Freundlich *n* value within the range of 1–10^30^. From Table 2, the *n* value was 2.304, which indicated efficient interaction between BRB species in solution and the ZnONPs adsorbent. The favorability of the dye adsorption process was further tested by the application of the Langmuir separation factor [R_L_ = (1/1 + K_L_C_o_)]^74^, where K_L_ is the Langmuir equilibrium adsorption constant^82^. The R_L_ value denotes a linear (R_L_ = 1), an irreversible (R_L_ = 0), a favorable (0 < R_L_ < 1) and unfavorable (R_L_ > 1) removal process^29^. The R_L_ value for BRB removal on ZnONPs was in the range of 0.115 to 0.394, which corroborates the favorable adsorption of BRB, indicating that ZnONPs are viable for the treatment of BRB polluted water. Besides, the monolayer uptake capacity of ZnONPs for BRB was 3.099 mg/g, which is higher than that of activated charcoal (0.081 mg/g)^85^ and polymeric gel (2.99 mg/g)^86^ but lower than 22.72 mg/g obtained for chitin nanoparticle^87^. Thus, the simple preparation procedure as well as potent antifungal properties (see discussion in the “Antifungal activity” section) would be the advantage in the application of ZnONPs for BRB wastewater treatment.

**Table 2:** The adsorption isotherm parameters for bromophenol blue adsorption onto ZnONPs.

Model	Parameter	Value
Langmuir	K_L_ (L/g)	0.154
	q_L_ (g/g)	3.099
	R^2^	0.9852
	SSE	0.5219
Freundlich	N	2.304
	K_F_ (L/kg)	0.639
	R^2^	0.9984
	SSE	0.0002
Temkin	B(g/g)	0.6495
	A (L/kg)	1.720
	R^2^	0.9799
	SSE	0.0368
Flory–Huggins	n_FH_	− 2.048
	K_FH_	0.0027
	R^2^	0.9854
	SSE	0.0075

### Kinetics and thermodynamics of adsorption

**Figure 5:**
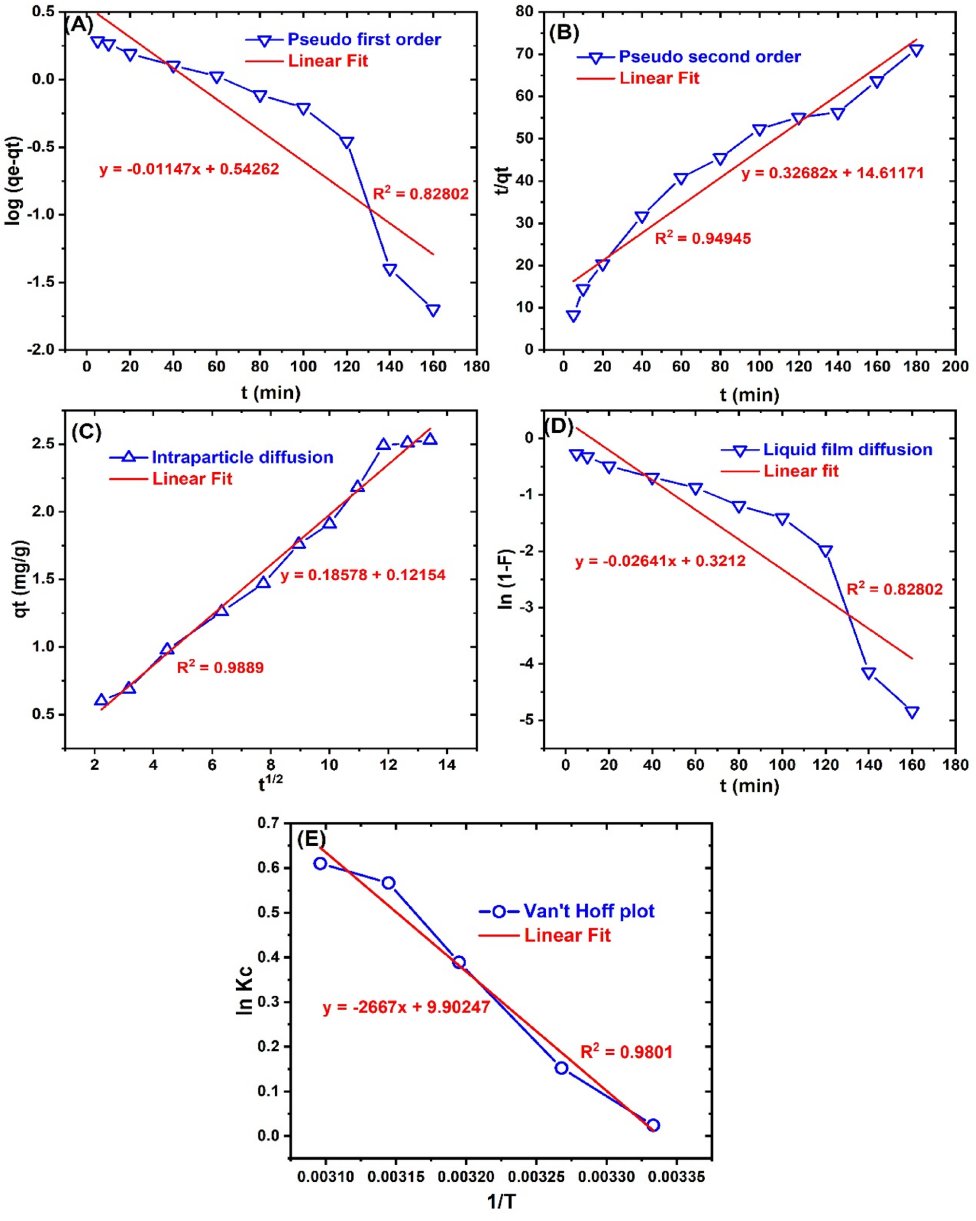
The (**A**) Pseudo-first-order, (**B**) Pseudo-second order, (**C**) Intraparticle diffusion, (**D**) liquid film diffusion and (**E**) Van’t Hoff plots for the adsorption of BRP onto ZnONPs.

The kinetic modeling of adsorption processes helps in the calculation of kinetic parameters, which is useful in system design and provides useful information on sorption mechanism^88^. The kinetics of BRB adsorption onto ZnONPs was modeled by the PFO, PSO, LFD, and IPD equations. The kinetic plots are presented in Fig. 5 while the obtained kinetic parameters are given in Table 3. It is obvious from the R^2^ of 0.9495 and the SSE of 0.7411 that the PSO was more suited than the PFO model in the kinetic description of BRB uptake onto ZnONPs. This was also supported by the closer calculated qe (3.0597 mg/g) of the PSO to the experimental qe (2.53 mg/g), than that presented by the PFO model (3.4883 mg/g). The best fit presented by the PSO models suggests the involvement of electrostatic interactions between the BRB molecules in solution and ZnONPs in the dye removal process^89,90^. This implication corroborates our deduction from the FTIR analysis obtained after BRB adsorption onto ZnONPs, which showed the involvement of electrostatic interactions. A similar result was reported in the adsorption of methylene blue onto ZnONPs impregnated sawdust based cellulose nanocrystals^91^.

**Table 3:** The kinetic parameters for bromophenol blue adsorption onto ZnONPs.

Model	Parameter	Value
Pseudo-first order	qe_(exp)_ (mg/g)	2.53
	K_I_ (min^-1^)	0.0264
	qe_(cal)_ (mg/g)	3.4883
	R^2^	0.8280
	SSE	0.7569
Pseudo-second-order	K_2_ (g/mg min)	0.0073
	qe_cal_ (mg/g)	3.0597
	R^2^	0.9495
	SSE	0.7411
Intraparticle diffusion	K_d_ (mg/g min^−1/2^)	0.1858
	C	0.1215
	R^2^	0.9889
	SSE	0.0581
Liquid film diffusion	K_fd_	0.0264
	Y	0.3212
	R^2^	0.8280
	SSE	4.0134

Furthermore, the LFD and IPD models provide reliable information about the diffusion mechanism of adsorption^62^. Comparing the two diffusion models, it is evident that the IPD model was best fitted to the diffusion process of BRB molecules onto ZnONPs as inferred from the high R^2^ (0.9889) and low SSE (0.0581). This indicates that the diffusion of BRB through the surface pores of ZnONPs played a more vital role in the overall adsorption process than the boundary layer diffusion. However, the occurrence of the intercept (0.1215), showed that particle diffusion was not solely responsible for BRB uptake, but to some extent involves film diffusion^30,49^. A similar finding in the adsorption of BRB onto *Solanum tuberosum* peel—silver nanoparticle hybrid was reported in our previous work^8^. The ultra-sonication applied prompted potent interaction between the dye species and the particle pores thus enhancing the particle diffusion mechanism. In addition, this observation is in line with the pore and SEM analysis, which showed a porous nature of ZnONPs that, would enhance the dye uptake from the solution.

The thermodynamics of BRB adsorption on ZnONPs was also evaluated from the Van’t Hoff plot as illustrated in Fig. 5E. The calculated thermodynamic parameters such as the changes in enthalpy (∆H^o^), entropy (∆S^o^), and free energy (∆G^o^) were used to analyze the spontaneity, randomness, and the physical or chemical nature of BRB uptake onto the synthesized ZnONPs^92^. The thermodynamic parameters obtained are presented in Table 4.

**Table 4:** Thermodynamic parameters for bromophenol blue adsorption onto ZnONPs.

Temp (K)	K_c_	ΔG^o^ (kJ/mol)	ΔH^o^ (kJ/mol)	ΔS^o^ (J/(mol K))	R^2^
300	1.024	− 0.059	22.173	74.02	0.9801
306	1.165	− 0.387			
313	1.475	− 1.012			
318	1.762	− 1.498			
323	1.841	− 1.638			

It is evident that the adsorption of BRB on the prepared ZnONPs adsorbent is spontaneous as the ∆G^o^ values were negative at all temperatures^93^. Again, the increase in negativity of the ∆G^o^ values with temperature increase shows that high temperature is favorable for BRB uptake^94^. Also, the positive ∆H^o^ value recorded is a clear indication of the endothermic nature of the dye adsorption on ZnONPs^95^, which corroborates increasing BRB uptake with temperature increase (Fig. 3e). This result is consistent with other studies depicting the endothermic adsorption of As(III)^29^, azo dyes^30^ and, Pb(II) ions^83^ on ZnONPs. It is important to mention that ∆H^o^ values in the range of 2.1–20.9 kJ/mol and 80–200 kJ/mol are attributed to physisorption and chemisorption respectively^96^. The ∆H^o^ of 22.173 kJ/mol obtained for the removal was slightly higher than the physisorption range but far lower than the chemisorption range. This indicated physicochemical adsorption of BRB onto ZnONPs rather than a purely chemical or physical uptake, dominated mainly by the physical forces of adsorption^97^. Again, this implies that a much lower energy barrier is to be overcome in the desorption of the BRB loaded ZnONPs during the regeneration of the adsorbent, compared to adsorption processes dominated or controlled by chemisorption. Furthermore, the increasing randomness at the ZnONPs-BRB solution interface was indicated by the positive ∆S^o^ value of 74.02 J/(mol K)^98^. This increasing random interaction at the interface must have enhanced efficient interaction between BRB molecules and the particle pores of ZnONPs, which accounted for the dominant role played by intraparticle diffusion in the overall adsorption process. Such increasing randomness is well documented in the adsorption of dyes onto ZnONPs based adsorbents^47,99–101^.

### Antifungal activity

**Figure 6:**
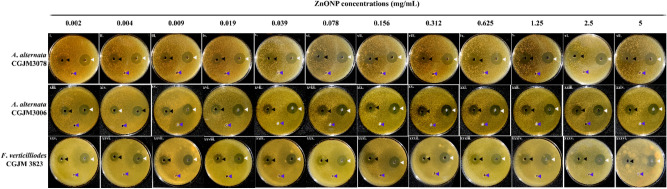
Plates representing the inhibition zones across a concentration range of ZnONPs against the fungal strains *Alternaria alternata* CGJM3078 (i–xii), *A. alternata* CGJM3006 (xiii–xxiv) and *Fusarium verticilliodes* CGJM 3823 (xxv–xxxvi). Black arrow heads indicate antimicrobial activities caused by ZnONPs, white indicates the bleach positive control, and blue indicates the sterile distilled water.

The antifungal activity of the ZnONPs was tested for possible application as simultaneous BRB removal and antifungal treatment of wastewater. Noticeably, as shown in Fig. 6, all of the tested concentrations of ZnONPs showed inhibitory effects on the three cultures, and the zones of inhibition increased consistently as the concentration of ZnONPs increased (Table 5). The strain *A*. *alternata* CGJM 3076 showed maximum susceptibility towards ZnONPs (0.002–5 mg/mL), as depicted by the largest zone of inhibition range from 25.09 to 36.28 mm. This was followed by *F*. *verticilliodes* CGJM 3823 (range from 23.77 to 34.77 mm) and *A. alternata* CGJM3078 (range from 22.73 to 30.63 mm). Concurrently, all the positive controls [bleach (5%)] exhibited similar diameter in their zones of inhibitions than those of ZnONPs, while none of the negative control (sterile water) showed effects**.**

**Table 5:** Antifungal activities (in vitro) of ZnONPs against the filamentous fungi *Alternaria alternata* (CGJM3078 andCGJM3006) and *Fusarium verticilliodes* CGJM 3823 are indicated in the form of inhibition zone sizes for each concentration of ZnONPs treatment.

ZnONPs (mg/mL)	Zone of Inhibition (mm)					
	*Alternaria alternata* CGJM3078	Bleach (5%) (positive control)	*Alternaria alternata* CGJM3006	Bleach (5%) (positive control)	*Fusarium verticilliodes* CGJM 3823	Bleach (5%) (positive control)
0.002	22.73	37.68	25.09	39.80	23.77	36.58
0.004	23.91	38.56	28.13	38.91	25.57	35.82
0.009	25.06	35.88	29.09	37.64	25.58	36.15
0.019	25.98	39.18	29.17	37.81	26.98	36.12
0.039	27.23	36.47	29.49	38.39	26.99	37.08
0.078	28.55	35.32	31.23	38.51	27.73	35.94
0.156	28.92	34.91	32.17	39.56	28.45	35.60
0.312	29.19	35.98	32.19	37.33	29.00	35.38
0.625	29.82	34.29	33.04	39.66	29.09	35.83
1.25	29.98	33.67	33.08	38.32	31.06	35.28
2.5	30.32	33.42	34.36	37.71	31.80	38.44
5	30.63	37.26	36.28	40.93	34.77	37.00

Bleach served as the positive control that exhibited similar diameter in their zones of inhibitions. The negative controls all did not show any activity.

Significantly less volumes of ZnONPs (40 µL) were used against all the tested fungi in this study in comparison to a previous study^33^, where 100 µL volume was implemented for an identical concentrations range (0.002–5 mg/mL). The enhanced antifungal efficiency of the formulated ZnONPs in the current study is possibly based on factors such as the size and shape of the particles, a large surface area to volume ratio that improved the solubility of the nanoparticles in comparison to the larger ones, the generation of reactive oxygen species (ROS) and the effective release of Zn^2+^ ions^51^.

**Figure 7:**
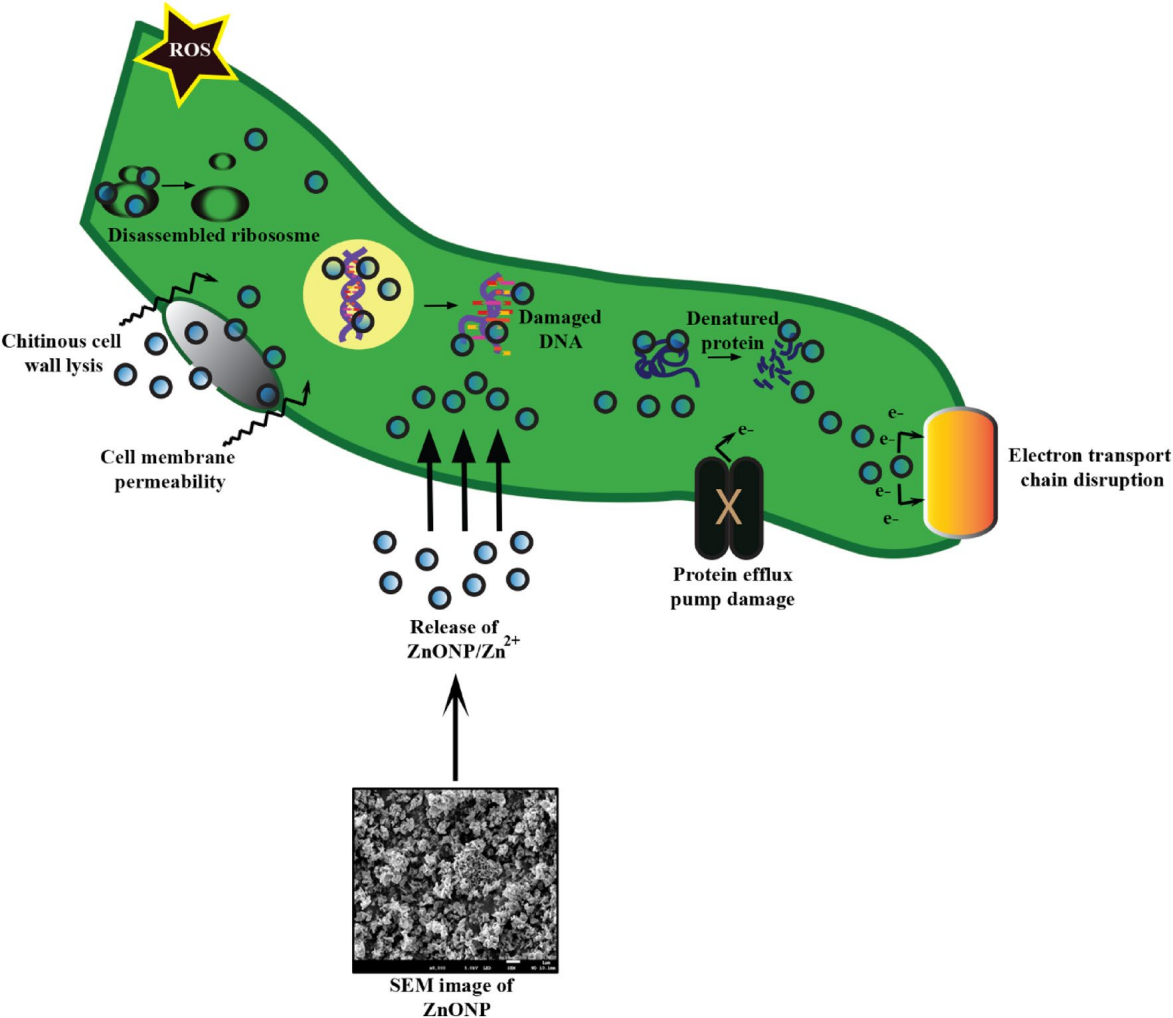
Schematic representation of the possible mechanism causing the antifungal ability of ZnONPs on a single fungal cell.

The exact underlying molecular mechanisms of ZnONPs antifungal activities are yet to be elucidated^102^. In this study we proposed a few plausible mechanisms (Fig. 7). Zn^2+^ can possibly be released from the surface of ZnONPs, as it has been shown in AgNPs^103^ that interacted with the fungal cell wall, passed and accumulated in the cytoplasm. This causes cell metabolism disturbances, impairment of the nucleic acid material by their irreversible adherence, ribosome disassembly, protein denaturations, electron chain disruptions, all of which ultimately resulting in cell death (Fig. 7). The interactions may also cause deformed fungal hyphae with ruptures and unusual bulging as was observed in an SEM study of antifungal activities of Zn compounds against pathogenic fungal strains of *Fusarium graminearum*, *Penicillium citrinum* and *Aspergillus flavus*^104^. The generation of the reactive oxygen species (ROS) (Fig. 7) as per a previous study^51^, can cause lipid peroxidation, leading to cell death. Simultaneously, the fungal cell wall can become more permeable because of the Zn^2+^, resulting in subsequent leakage of the plasma fluid and cellular organelles causing cellular senescence (Fig. 7). SEM structure imaging of the ZnONPs developed in the present study revealed the average size of ZnONPs as 65.3 nm. This is consistent with the previous findings^31^, where the average size was reported as 70 ± 15 nm. Those ZnONPs successfully inhibited the growth of mycotoxin producing fungi, *Botrytis cinerea* and *Penicillium expansum*, causing cellular perturbations, and fungal hyphal distortion.

## Conclusions

Zinc oxide nanoparticles (ZnONPs) were synthesized in a- simple one-pot system and applied for the adsorption of bromophenol blue (BRB) and as an antifungal agent against *Alternaria alternata* and *Fusarium verticilliodes* filamentous fungi. The FTIR showed the presence of the O–H and Zn–O bands on the synthesized ZnONPs, which were responsible for the adsorption of BRB via electrostatic, hydrophobic, and weak Vander Waals interaction. The XRD, UV, and EDX characterizations showed the successful synthesis of the ZnONPs. Thermal analysis revealed high thermal stability of the ZnONPs with a 22.92% weight loss at 800 °C, while BET analysis showed sufficient surface area and pore properties desirable for efficient BRB uptake from solution. The SEM and TEM morphology displayed an irregular shaped and aggregated porous structure of ZnONPs. The solution temperature, pH, time, BRB concentration, and ZnONPs dosage were found to influence significantly the BRB uptake on ZnONPs. The operating conditions were selected as pH, temperature, time, dosage, and concentration of 4.0, 300 K, 180 min, 0.1 g, and 50 mg/L respectively. The Freundlich model presented the best fit to the isotherm analysis for the adsorption process compared to the Langmuir, Flory Huggins, and Temkin isotherm models. For kinetic analysis of BRB adsorption onto ZnONPs, the pseudo-second-order and intra-particle-diffusion were better fitted than the Pseudo-first-order and liquid-film-diffusion models. A feasible, spontaneous, random, and endothermic removal of BRB onto ZnONPs was revealed from the thermodynamic evaluation of the process. The synthesized ZnONPs were found to be efficient in the adsorption of BRB from solution with a maximum monolayer uptake capacity of 3.099 mg/g, which was higher than some previously reported adsorbents.

In addition, the prepared ZnONPs exhibited potent antifungal activities against the filamentous fungi, *A. alternata* and *F. verticilliodes*. The antifungal effects, such as inhibition of growth and reproduction of the filamentous fungi, are enhanced significantly with the ZnO nanostructures. Therefore, the present study, together with previous studies, showed that ZnONPs have tremendous potential as an effective postharvest disease control antifungal agents against filamentous fungi, or even possibly in the field. No studies have, however, tested this in vivo.

## Supplementary Information

Supplementary Information.
