# Supplementary material for: One-pot synthesis of zinc oxide nanoparticles via chemical precipitation for bromophenol blue adsorption and the antifungal activity against filamentous fungi
Source: Sci Rep. 2021 Apr 15;11:8305. doi: 10.1038/s41598-021-87819-2 (PMC8050082; doi:10.1038/s41598-021-87819-2)
Supplement: Supplementary file 1 — Supplementary Information. [file 41598_2021_87819_MOESM1_ESM.docx]

**One-pot synthesis of zinc oxide nanoparticles via chemical precipitation for bromophenol blue adsorption and the antifungal activity against filamentous fungi**

**Kovo G. Akpomie^1,2^, Soumya Ghosh^3^, Marieka Gryzenhout^3^, Jeanet Conradie^1^**

^1^Department of Chemistry, University of the Free State, Bloemfontein, South Africa.

^2^Department of Pure & Industrial Chemistry, University of Nigeria, Nsukka, Nigeria.

^3^Department of Genetics, University of the Free State, ZA9300, Bloemfontein, South Africa.

E-mail: *[kovo.akpomie@unn.edu.ng](mailto:kovo.akpomie@unn.edu.ng);

**SUPPLEMENTARY INFORMATION**

**Adsorption isotherm modeling**

The isotherm modeling of BRB adsorption onto the prepared ZnONP was studied by the Langmuir, Freundlich, Temkin and Flory Huggins models, given respectively ^1^:

$$\frac{C_{e}}{q_{e}}=\frac{1}{q_{L}K_{L}}+\frac{C_{e}}{q_{L}} (1)$$

$$\log q_{e}= \log K_{F}+ \left( \frac{1}{n} \right)\log C_{e} (2)$$

$$q_{e}=B\ln A+B\ln C_{e} (3)$$

$$Log\frac{\theta}{C_{O}}=LogK_{FH}+n_{FH}Log\left( 1-\theta\right) (4)$$

Where *K_L_* (L/g) is the Langmuir constant corresponding to the bonding energy, *q_L_* (g/g) is the maximum monolayer adsorption capacity, C_o_ (g/L) represents the initial BRB concentration, while Ce (g/L) is the BRB concentration at equilibrium. The Freundlich parameter *K_F_* (L/kg) is the sorption intensity, *n* is the adsorption capacity, while the Temkin constants *A* (L/g), and B corresponds to the binding energy and heat of adsorption respectively. The symbols *n_FH_* and *K_FH_* are the Flory Huggins constants and θ = [1 – (C_e_/C_o_)] represents the uptake surface coverage.

**Sorption kinetics**

The process kinetics of BRB adsorption onto ZnONP was study by the Pseudo-first-order , Pseudo-second-order, intraparticle diffusion and Liquid film diffusion models given respectively ^2^:

$$\log\left( q_{e}-q_{t} \right)=\log q_{e}-\frac{K_{1}}{2.303}t (5)$$

$$\frac{t}{q_{t}}=\frac{1}{K_{2}{q_{e}}^{2}}+\frac{t}{q_{e}} (6)$$

$$q_{t}=K_{d}t^{\frac{1}{2}}+C (7)$$

$$Ln\left( 1-F \right)=Y-K_{FD}t (8)$$

Where *qt (*g/g) is the ZnONP adsorption capacity at a time *t* (min), *K_I_*_,_ (min^-1^), K_2_ (g/g min), K_d_ (g/g min^-1/2^) and K_FD_ are the rate constants of the pseudo-first order, pseudo-second-order, intraparticle-diffusion and liquid film diffusion models respectively. The intercept (C) of the ID model depicts the presence of boundary layer diffusion, while *F* represents the fractional equilibrium attainment of the liquid film diffusion model.

**Sorption thermodynamics**

The thermodynamic parameters of BRB uptake onto the synthesized ZnONP were determined to evaluate the spontaneity, disorderliness, and nature of adsorption by the application of the following equations ^3^:

$$\Delta G^{0}= -RT \ln K_{c} (9)$$

$$\ln K_{C}=-\left( \frac{\Delta H^{0}}{RT} \right)+\left( \frac{\Delta S^{0}}{R} \right) (10)$$

Where ∆H^o^, ∆G^o^, and ∆S^o^ depicts the enthalpy, free energy, and entropy changes, respectively, K_c_ is the sorption equilibrium constant, T (K) is the absolute temperature and R (8.314 J/mol K) is the universal gas constant.

**References**

1. Diraki, A., Mackey, H., McKay, G. & Abdala, A. A. Removal of oil from oil–water emulsions using thermally reduced graphene and graphene nanoplatelets. *Chem. Eng. Res. Des.* **137**, 47–59 (2018).

2. Ezekoye, O. M. *et al.* Biosorptive interaction of alkaline modified Dialium guineense seed powders with ciprofloxacin in contaminated solution: central composite, kinetics, isotherm, thermodynamics, and desorption. *Int. J. Phytoremediation* **22**, 1028–1037 (2020).

3. Akpomie, K. G., Onyeabor, C. F., Ezeofor, C. C., Ani, J. U. & Eze, S. I. Natural aluminosilicate clay obtained from south-eastern Nigeria as potential sorbent for oil spill remediation. *J. African Earth Sci.* **155**, 118–123 (2019).
